# Factors impacting the complexity of the leporid intracranial joint

**DOI:** 10.1111/joa.70031

**Published:** 2025-08-07

**Authors:** Amber P. Wood‐Bailey, Heather E. White, Samuel R. R. Cross, Nathan S. Jeffery, Philip G. Cox, Alana C. Sharp

**Affiliations:** ^1^ Department of Musculoskeletal and Ageing Science, Institute of Life Course and Medical Sciences University of Liverpool Liverpool UK; ^2^ Department of Life Sciences Natural History Museum London UK; ^3^ Department of Cell and Developmental Biology University College London London UK

**Keywords:** complexity analysis, cranial morphology, geometric morphometrics, lagomorphs, locomotion, sutures

## Abstract

Leporid lagomorphs, the rabbits and hares, exhibit unique cranial traits that distinguish them from their closest relatives, the Ochotonidae (pikas), and all other mammals. Among these features, the intracranial joint stands out as the only example of cranial kinesis in mammals and is hypothesised to dissipate kinetic energy during high‐speed locomotion. Despite its potential functional importance, the morphology of the joint remains understudied. High complexity in other cranial sutures has been associated with behavioural traits such as feeding and head butting. In this study, we quantified the complexity of the intracranial joint using two independent metrics and explored its relationships with overall cranial shape, locomotor mode, cranial size and body mass, burrowing habit, and facial tilt angle. We found significant correlations between complexity and locomotor mode as well as with facial tilt angle, indicating that cursorial species have less complex sutures and highlighting a potential link between the complexity of suture interdigitation and facial tilting. However, complexity did not correlate with size or burrowing habit. Our findings shed more light on the functional anatomy of the leporid cranium and emphasise the need for further research on ontogenetic development, biomechanics, and behaviour to fully understand the evolutionary and functional significance of these unique cranial traits.

## Graphical abstract

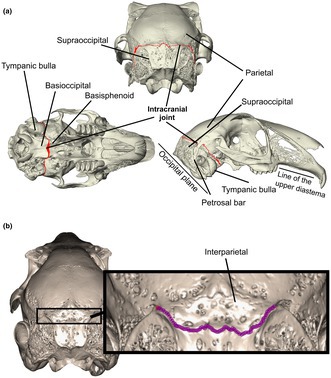

## 1 INTRODUCTION

Leporid lagomorphs, the rabbits and hares, exhibit a variety of morphological traits that distinguish them from their closest living relatives, the pikas (*Ochotona*): proportionally longer hind limbs, larger pinnae, a generally larger body size, and several cranial traits including fenestration of the lateral maxilla, anterior facial tilting, and an intracranial joint (ICJ) (Bramble, 1989; Kraatz et al., 2015; Moss & Feliciano, 1977; Wood‐Bailey et al., 2022; Wood‐Bailey & Sharp, 2025). Despite their unique morphology, lagomorphs are understudied in comparison to their rodent relatives, particularly in relation to the cranium. To some extent, this is due to the conserved nature of the order, whereas rodents show remarkable taxonomic, morphological, and ecological diversity.

It seems likely that at least some of the unique traits in the lagomorph cranium are functionally related to locomotion (Bramble, 1989; DuBrul, 1950; Kraatz et al., 2015). The most pronounced phenotypic variation among lagomorphs is the way in which they move. Some adopt a scrambling, generalist gait (e.g., *Brachylagus*), some have a hopping‐based, saltatorial gait that averages around 20–30 km/h (e.g., *Oryctolagus*) and some have a specialised, cursorial gait where top speeds may reach 75 km/h, with an acceleration speed of 4.4 m/s^2^ (Carrier, 1995) and a vertical jump height of 4.5 m (e.g., *Lepus*). Species that run at high speeds tend to have more acute facial tilting (Kraatz et al., 2015), a feature characterised by ventral deflection of the rostrum relative to the occipital bone, more extensive fenestration in the maxilla (DuBrul, 1950), and potentially, a functionally kinetic cranium at the ICJ (Bramble, 1989).

**FIGURE 1:**
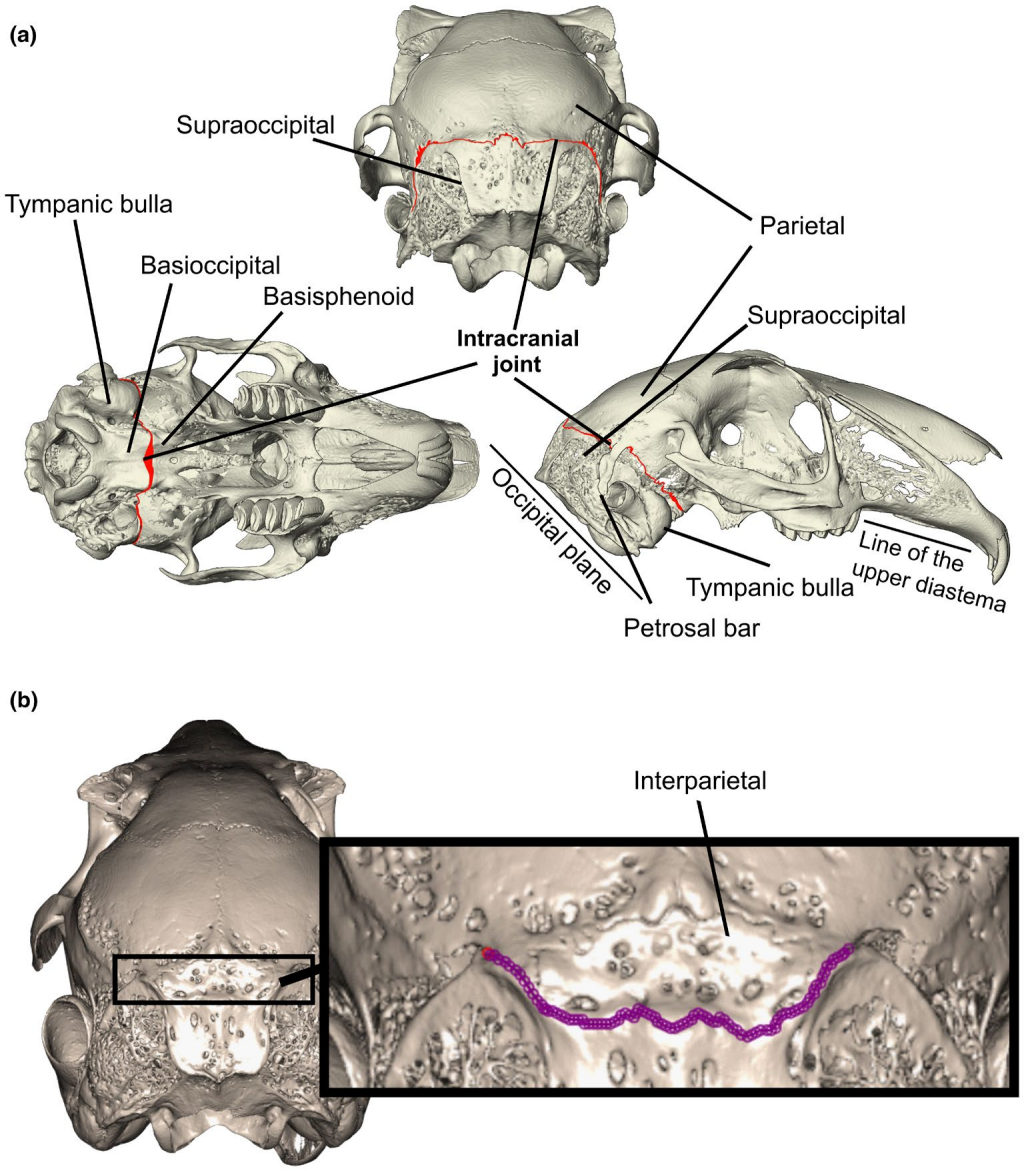
Anatomy of the intracranial joint and the 2D semi‐landmarks used to capture complexity in the intracranial joint. (a) The intracranial joint can be seen here (in red) between the parietal and occipital dorsally and the basioccipital and basisphenoid ventrally. In hares (as in this figure) the interparietals are fused to obliteration. In several rabbit genera (*Oryctolagus, Bunolagus and Sylvilagus*), where the interparietals remain unfused, the joint is diverted around the posterior edge of the interparietal (as labelled in b). This figure is adapted from Wood‐Bailey et al. (2022). (b) Landmarks were placed manually and resampled to 250 per specimen.

The ICJ appears in the leporid branch of the lagomorphs, but it is absent in the sister family Ochotonidae (Wood‐Bailey et al., 2022). It is located between the parietal and occipital bones dorsally, the basioccipital‐basisphenoid ventrally, and between the squamosal and otic complex laterally, dividing the cranium into discrete anterior and posterior units (Figure 1). This location, developmentally, is at the junction between bones of the anterior skull that undergo intramembranous ossification and bones of the posterior skull that undergo endochondral ossification (Kyomen et al., 2023). The dorsal‐most portion of the joint is composed of the lambdoidal suture, a suture of unknown developmental origin (White et al., 2021), which forms the fibrous connection between the parietal and occipital bones. In leporids, this suture appears to remain patent throughout life, allowing a degree of movement at the basioccipital‐basisphenoid articulation (Bramble, 1989). In hares, the fusion of the interparietal with the parietals at the midline allows for the ICJ to form a straighter line compared with other genera where the interparietal is unfused (such as in rabbits). However, these differences in morphology and potential function of the ICJ between rabbits and hares have not been explored quantitatively.

Suture complexity, which refers to the geometric intricacy of the sutural line, or the deviation from a straight line (Allen, 2006; White et al., 2020), has been correlated in a range of taxa with various biomechanical demands: for example, locomotion (Alheit et al., 2022), mastication (Sun et al., 2004) and other activities such as sparring (Curtis et al., 2013). Complexity analysis is increasingly used to compare the differences in interdigitation in cranial sutures between taxa (Buezas et al., 2017; Byron, 2009; White et al., 2020). Sutures facilitate craniofacial growth by providing growth sites (Jin et al., 2016), allowing for the expansion of the cranium to permit brain development (White et al., 2020), and they provide a key biomechanical function by absorbing and/or dissipating forces (Curtis et al., 2013; Sharp et al., 2023). It has been demonstrated that cranial bone with sutures absorbs between 16 and 100% more energy per unit volume than bone without sutures due to increased collagen‐filled surface area (Jaslow, 1990). Fractal interdigitations, formed as a response to loading, increase this surface area (Herring, 2008) and therefore, variation in interdigitation frequency and magnitude corresponds to variation in force attenuation ability (Herring, 2008). Sutures also dissipate forces across the cranial bones due to viscoelastic tissue properties (Herring & Teng, 2000; Ross et al., 2018), the extent of which is likely affected by the properties of the bone surrounding the sutures (Farke, 2008). It has been hypothesised that the ICJ in leporids allows relative motion between the posterior and anterior units of the cranium, providing shock absorption and reducing the impulsive load experienced by the anterior unit and eyes upon impact during galloping (Bramble, 1989). As the ICJ comprises the lambdoidal suture as its proposed hinge, quantifying the complexity of this suture may reveal insights into the function of the joint.

Whilst locomotor mode likely influences leporid cranial morphology (Bramble, 1989; DuBrul, 1950; Kraatz et al., 2015; Kraatz & Sherratt, 2016; Moss & Feliciano, 1977; Watson et al., 2021) and may correlate with ICJ complexity, other factors could be important covariates, including phylogeny, body size, and other behavioural traits like burrowing. A strong phylogenetic signal in cranial shape has been identified in several mammalian taxa, including lagomorphs (Alhajeri & Steppan, 2018; Cardini & Elton, 2008; Radinsky, 1984). Size also plays an important part in cranial morphological variation (Cardini & Polly, 2013; Porto et al., 2013) and, whilst lagomorphs have been size‐constrained throughout evolution, extant taxa vary in body size by over an order of magnitude. The smallest species are around 100–300 g (*Ochotona princeps*) whilst the largest species can attain sizes of 3000–4000 g (*Lepus arcticus*; see Data S1). Furthermore, several leporid lagomorphs are adept burrowers, in particular, the European rabbit (*Oryctolagus cuniculus*). The effect of burrowing on skull shape in mammals has been identified (Gomes Rodrigues et al., 2016; McIntosh & Cox, 2016a, 2016b) and a number of studies have looked at the relationship between burrowing, locomotion, and cranial traits in lagomorphs (Kraatz & Sherratt, 2016; Martin et al., 2022) but we do not yet know to what extent this behaviour influences ICJ complexity or function.

There are two primary aims of this work: first, to quantify complexity in the lagomorph ICJ; and second, to compare complexity scores with covariates such as overall cranial shape and size, body mass, burrowing habit, locomotor mode, and facial tilt.

We hypothesise that if the ICJ is functionally acting as a mobile joint to dissipate locomotor forces:

The degree of complexity will differ significantly between locomotor modes.

If the role of the ICJ is primarily in force attenuation and/or dissipation, we expect to see variation between groups depending on locomotor mode. To test this, we will quantify complexity and use analysis of variance (ANOVA) and phylogenetic analysis of variance (PANOVA) to test the equality of means between groups.

The degree of complexity will vary with differing facial tilt angles.

Facial tilt angle has been found to vary with locomotion and, if H1 is supported, we expect there will be a correlation between facial tilt angle and complexity. We will test quantified complexity covariation with facial tilt via phylogenetic generalised least squares (PGLS). Linked to this, we expect that shape (particularly PC1) will be associated with morphological changes linked to facial tilting (such as doming of the skull), as found by Kraatz and Sherratt (2016). Therefore, we hypothesise that any correlation found between facial tilt angle and complexity, will also be the case for cranial shape.

There will be no correlation between complexity and cranial size, body mass or burrowing.

Leporids are relatively conservative in size (compared with, for example, rodents; Tomiya & Miller, 2021). Leporids on the larger end of the spectrum are found in all locomotor categories, so we can postulate that mass will not show a relationship with locomotor mode. Furthermore, burrowing in lagomorphs is limb‐dominated, and therefore, there is little reason to believe that this behaviour would impact cranial morphology in lagomorphs in terms of function (Hildebrand & Hurley, 1985). However, recent work suggests that burrowing behaviour in leporids does predict brain size variation (Todorov et al., 2022). These assumptions will be tested with ANOVA and PGLS.

## 2 METHODOLOGY

### 2.1 Specimens and scan preparation

We obtained high‐resolution micro‐computed tomography scans (μCT) of individuals from 14 species representing 91% of extant leporid genera and ~20% of extant species (Burgin et al., 2018). All specimens were adults, as determined by tooth eruption. Due to the high number of mono‐specific genera, we wanted to avoid oversampling speciose genera. Among these are *Lepus* and *Sylvilagus*, both of which include numerous species. However, we acknowledge that omitting many species may be a limitation. Furthermore, a single specimen was used for each species; this is due to the lack of high‐resolution CT data available for the species used, many of which are rarely studied. For the most speciose taxon *Lepus*, three cursorial and two non‐cursorial species were sampled to account for a variety of running speeds (see Table S1 for a more comprehensive overview of species sampled and source). The μCT scans were segmented in Avizo 9.2.0 (Thermo Fisher Scientific, Waltham, MA, USA) using automatic thresholding and manual segmentation tools to reconstruct cranial morphology. All models used in analyses are publicly available, with details of acquisition parameters, on Morphosource (www.morphosource.org).

It should be noted here that recent research utilising ultra‐conserved elements to generate a phylogenetic tree has recommended the reclassification of *Brachylagus idahoensis* as within the genus *Sylvilagus* (Cano‐Sánchez et al., 2022). However, to account for phylogeny in our analyses, we used a phylogenetic tree that we published previously using morphological characters (Wood‐Bailey et al., 2022). This tree refers to *Brachylagus* as a sister‐taxa to *Sylvilagus*. In terms of cranial morphology, *Brachylagus* is quite different from other members of the *Sylvilagus* genus because it is a dwarfed species. Therefore, for the sake of continuity with our published phylogeny and to acknowledge the morphological differences, we have referred to the species as *Brachylagus* in this manuscript.

### 2.2 Geometric morphometric analyses

**FIGURE 2:**
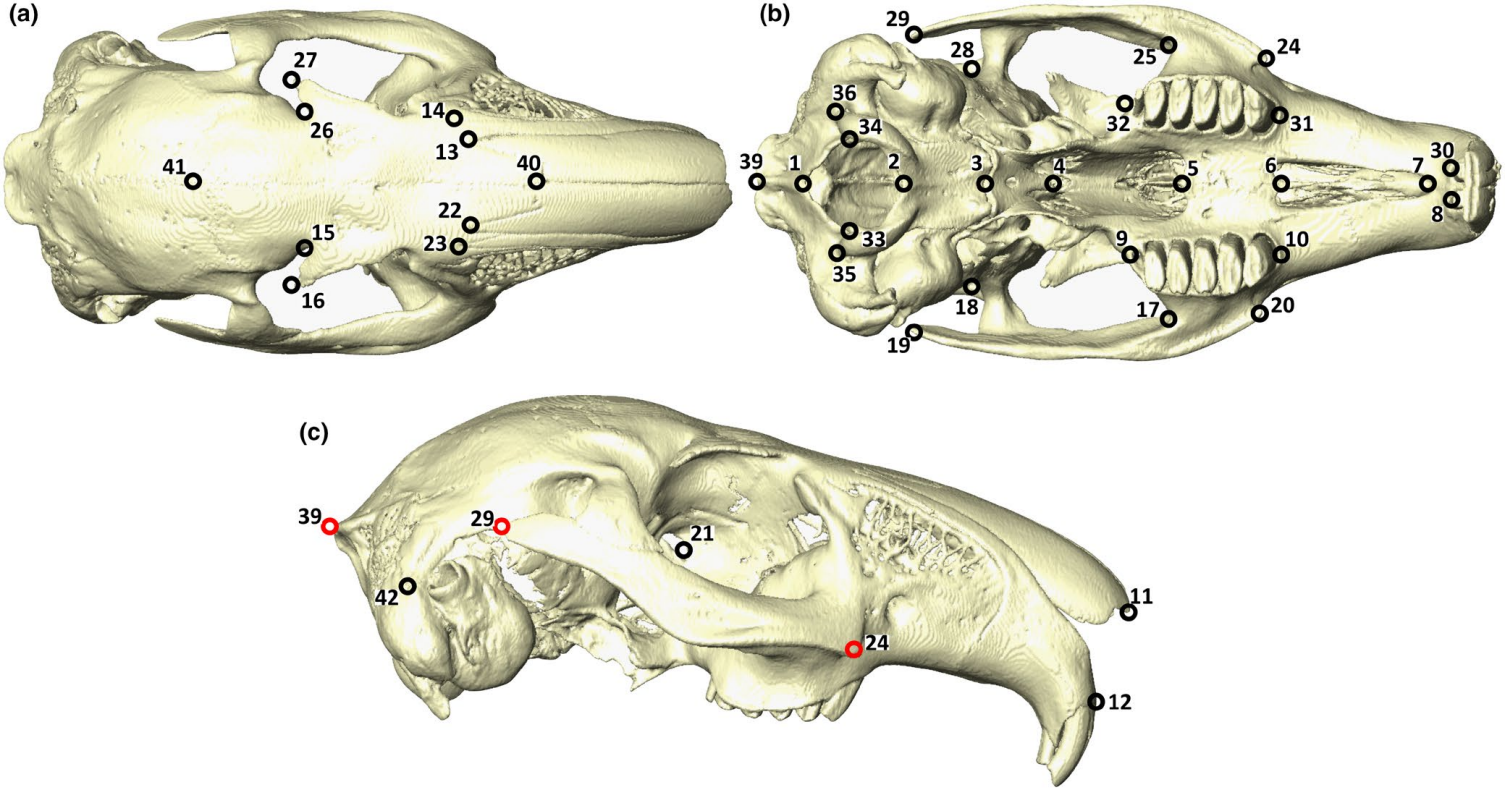
3D homologous anatomical landmarks used to quantify shape variation. (a) Dorsal view. (b) Ventral view. (c) Lateral view. See Table S2 for a description of all 43 landmarks. Landmarks in red are shown in multiple planes.

A total of 43, type I and II, 3D landmarks were placed on the cranial models in Avizo 9.2.0 (Figure 2; see Table S1–S2 for a comprehensive list of landmarks used). Landmarking was performed by a single user (AWB) to avoid interobserver error. Landmarks were chosen to account for the entire cranium and adapted from Kraatz and Sherratt (2016); however, we excluded the type III landmarks that were used to capture facial tilt angle to avoid over‐representing the dorsal braincase relative to the rest of the skull and because facial tilt was included as a single angular value in our analyses (see below). The function ‘*gpagen’* in the package *geomorph* (Adams et al., 2021; Baken et al., 2021) in the R statistical environment version 4.3.0 (R Core Team, 2022) was used to carry out generalised Procrustes analysis whereby landmarks were translated, rotated, and uniformly scaled to allow for the direct comparison of shape (Adams et al., 2021; Baken et al., 2021).

Having undergone Procrustes transformations, the resultant data depict the effects of shape in a multidimensional space. We then performed principal components analysis (PCA) of the transformed coordinates using the function ‘*gm.prcom*’ in the package *geomorph*, reducing the dimensionality of the data and identifying the percentage of variance accounted for by each principal component (Adams et al., 2021; Baken et al., 2021). The transformed coordinates returned by this PCA were then used as a variable in PGLS using the function ‘*procD.pgls*’ in the R package *geomorph* (Adams et al., 2021; Baken et al., 2021).

### 2.3 2D suture data collection

Each reconstructed μCT model was uniformly positioned in the R package *rgl* (Murdoch & Adler, 2022) to account for parallax. Images were then taken in an axial view, showing the posterior‐most portion of the skull to most effectively visualise the lambdoidal suture (Figure 1). A varying number of 2D semi‐landmarks (landmarks that are not specific to identifiable anatomical points) were then placed on the lambdoidal suture in the *StereoMorph* R package (Olsen & Haber, 2022). These landmarks were then resampled to an equidistantly spaced 250 per suture for all individuals and subjected to generalised Procrustes analysis, as above. We acknowledge that reducing an inherently three‐dimensional structure to two dimensions may reduce the full complexity of the picture of the suture (Remesz et al., 2023). However, the methodologies available by which to quantify suture complexity are currently limited to two dimensions. Furthermore, due to the lack of homologous anatomical landmarks available in sutures, it is difficult to apply traditional geometric morphometric methodologies.

### 2.4 Phylogeny

To account for the effect of evolutionary relationships on our data, we included a time‐scaled consensus tree derived from previous work (Wood‐Bailey et al., 2022; Figure S1). This phylogeny used morphological characters from previously published phylogenies (Meng et al., 2003), with new characters added representing unique cranial features in the leporid lagomorphs and divergence time estimates based on previously published molecular phylogenies.

### 2.5 Locomotor categorisation

In the absence of data on maximum running speed for many leporids, each species was binned into three distinct categories following Kraatz and Sherratt (2016) (see Data S1). The three locomotor categories used were: generalised, saltatorial, and cursorial. Generalist species do not exhibit regular hopping, and the mode can be described as “scampering” (such as *Brachylagus*). Saltatorial species display significant jumping actions, and this is demonstrated in their limb anatomy (such as *Oryctolagus*). Saltatorial species are also relatively fast for their body size. Cursorial species are those that routinely run at high speed (such as many species within *Lepus*). Cursorial species also exhibit saltatorial behaviour at slower speeds but are categorised by the propensity for extremely high‐speed running. Though precise delimitation of locomotor groupings is often difficult (Chen & Wilson, 2015), the categories used here were intended to represent broad differences in mode, particularly in relation to maximal speed performance and incurred forces (which are of principal interest to this study), and crucially permitted categorisation of all leporid taxa, which is necessary for downstream analyses.

### 2.6 Facial tilt

For facial tilt, we took the median tilt angle for each species from the data presented by Kraatz et al. (2015). They defined facial tilt as the angle between the upper diastema and the occipital plane (as shown in Figure 1a). In addition, we measured facial tilt angle for a number of species not included in Kraatz et al. (2015): *Lepus arcticus, Lepus europaeus, Nesolagus timminsi, Pentalagus furnessi, Pronolagus rupestris*, and *Sylvilagus brasiliensis* (see Data S1). These angles were taken using the measurement protocol outlined in Kraatz et al. (2015), in Avizo. A Shapiro–Wilk test was performed on facial tilt to ascertain distribution normality. A *p‐*value of 0.418 was returned; thus, the data did not significantly depart from normality, and values were used untransformed in statistical analyses.

### 2.7 Body and cranial size

Since size has an influence on locomotor mode and function (Biewener, 1989; Cloyed et al., 2021) and cranial shape (Bertrand et al., 2016) in mammals in general, we included averages for the body mass of each species in the Data S1. Sexual dimorphism is not considered to be a significant factor in leporid body mass, so sex was not accounted for (Todorov et al., 2022). Body mass for each species used was collated from the literature available (see Data S1). In the case of *N. timminsi*, in the absence of any literature on body mass, an estimate was given by individuals who work with this rare species in the field (pers. comm. Tilker and Nguyễn, 2023). A Shapiro–Wilk test was performed on body mass to ascertain distribution normality. A *p‐*value of 0.8104 was returned; therefore, the data did not significantly depart from normality, and values were used untransformed.

Centroid size was also collected during the generalised Procrustes analysis. This is the square root of the sum of squared distances of each landmark from the centroid and gives a geometric size measurement that represents local, as opposed to just somatic, scaling effects, and is less labile to the modulation of skull weight by the formation of fenestrations (Bramble, 1989; Zelditch et al., 2012).

### 2.8 Burrowing

The burrowing habit variable was binarised based on descriptions from the literature (Bider, 1961; Flux, 1970; Gray, 1993; Jin et al., 2010; Kolb, 1985; Kraatz et al., 2015; Maheswaran, 2006; Nowak, 1999; Rachlow et al., 2005; Stavy et al., 1985; Velázquez & Guerrero, 2020; Yamada et al., 2000). We only included species that actively burrow, as opposed to co‐opting another animal's burrow or rearing young in a shallow depression. This is because it is the act of burrowing, as opposed to simply dwelling in burrows, that we would expect to directly influence morphological change.

### 2.9 Complexity analysis

There are a number of metrics that have been developed to quantify suture complexity (see White et al., 2020, for a comprehensive evaluation of these metrics). In this study, we selected two uncorrelated metrics to account for different aspects of complexity: sinuosity index (SI) and a short‐time Fourier transform with a power spectrum density (PSD) calculation. SI is a ratio between the length of the suture including all curves/interdigitations and the direct length of the suture from start to end (Byron, 2009; White et al., 2020). Fourier transform with PSD, analyses the frequency of a signal over short overlapping time intervals. It does this by converting shape data (e.g., Procrustes transformed landmarks) to a series of sinusoidal waves with different frequencies and amplitudes and then computing the distribution of power across different frequencies in the shape (Allen, 2006; White et al., 2020).

As described by White et al. (2020), these metrics do not correlate with one another closely, reflecting that they measure different components of complexity. It has been shown that SI is more sensitive to the number of interdigitations (i.e., SI classifies the sutures with the most interdigitations as the most complex). Given that interdigitation is only one aspect of suture complexity, we also computed the PSD score, which is sensitive to a wider range of complexity features such as irregularity as well as interdigitation (White et al., 2020). Both approaches (SI and PSD) were implemented on the Procrustes superimposed semi‐landmarks of the lambdoidal suture.

### 2.10 Phylogenetic generalised least squares

For the continuous data sets (facial tilt and body mass), PGLS was performed using the function'*gls'* in the R package *nlme*, where complexity scores for SI and PSD were coded as response variables and modelled as a function of the predictor variable (either facial tilt, centroid size or body mass; Pinheiro & Bates, 2022). The models accounted for phylogenetic heritage using the time‐scaled topology of Wood‐Bailey et al. (2022), under a Brownian model of evolution. We also conducted PGLS for shape and complexity using the function ‘*procD.pgls’* in the R package *geomorph* (Adams et al., 2021; Baken et al., 2021). Complexity scores for SI and PSD were inputted as predictor variables, and shape was used as the response variable.

### 2.11 Statistical comparisons of categorical variables

To assess phylogenetic signal (λ) in the residuals of the relationships between the two‐complexity metrics and locomotor mode, we used the function ‘*phylosig’* in the R package *phytools* (Revell, 2024). Both one‐way ANOVA and PANOVA were employed to statistically assess the equality of means in the complexity metrics versus locomotor mode using the ‘*anova_test*’ function in the R package *rstatix* (Kassambara, 2023), and the ‘*phylANOVA*’ function in the package *phytools* (Revell, 2024). ANOVA was also employed to assess complexity and burrowing habit.

## 3 RESULTS

### 3.1 Complexity

The complexity analysis reveals a normally distributed range of values for each metric (SI and PSD). However, there is disagreement between metrics on where species fit on the scale of most complex to least complex, given the metrics capture differing components of complexity (see Data S1 for values for individual specimens). The species with the most complex suture according to SI is *Caprolagus hispidus*; for PSD, it is *Nesolagus timminsi*. The species with the least complex suture according to SI is *Pronolagus rupestris*; for PSD, it is *Lepus capensis*. Whilst there is considerable difference in metric scores between species, the scores are relatively low when compared with the scores for cranial sutures across mammals as a whole (White et al., 2020).

### 3.2 Locomotor mode

The phylogenetic signal (*λ*) within the relationships between the two‐complexity metrics and locomotor mode was tested, and it was found that the relationship between the PSD metric and locomotor mode has a high phylogenetic signal (*λ* = 0.983). This suggests that PANOVA is a suitable model to assess the significance of this relationship. However, the phylogenetic signal in the relationship between the SI metric and locomotor mode is low (*λ* = <0.001), suggesting that ANOVA is an appropriate model to test this relationship.

For PSD, there was no statistical difference between locomotor modes (*p = 0.082*; Table 1). Post‐hoc pairwise comparisons did not find any significant differences between the groups. For SI, there was a statistically significant difference between locomotor modes (*F =* 5.0820, *p* = 0.027). Post‐hoc pairwise comparisons found that generalists and saltators were significantly different from one another (*p* = 0.0350), but cursors and generalists (*p =* 0.05) and cursors and saltators (*p =* 0.9670) were not (Table 1).

**TABLE 1:** ANOVA and PANOVA results for the suture complexity metrics versus locomotor group.

MODEL (~ LOCO. GRP.)	*F*	*p*	Η^2^G	*λ*	C V G	C V S	G V S	Pref.
PSD	6.316	0.015	0.535	−	0.019	0.618	0.035	
		*0.082*		*0.983*	*0.192*	*0.629*	*0.087*	*
SI	5.082	0.027	0.48	−	0.058	0.967	0.035	*
		*0.129*		*< 0.001*	*0.428*	*0.908*	*0.096*	

*Note*: ANOVA results are presented on the upper row and (*F*), significance value (*p*) and the estimated effect size (η^2^G), as well as post‐hoc pairwise comparisons of significance between the three locomotor groupings (G = generalised, S = saltatorial, C = cursorial). Differences in significance (main and pairwise) resulting from the PANOVA are presented on the lower in italics alongside the measure of phylogenetic signal (Pagel's *λ*) within the relationship. Note that for the SI metric, *λ* is low and therefore standard ANOVA is the more appropriate model since this does not assume a Brownian mode of evolution (which PANOVA does). We have indicated the most suitable model for each relationship with a * in the preferred column.

### 3.3 Facial tilt

**FIGURE 3:**
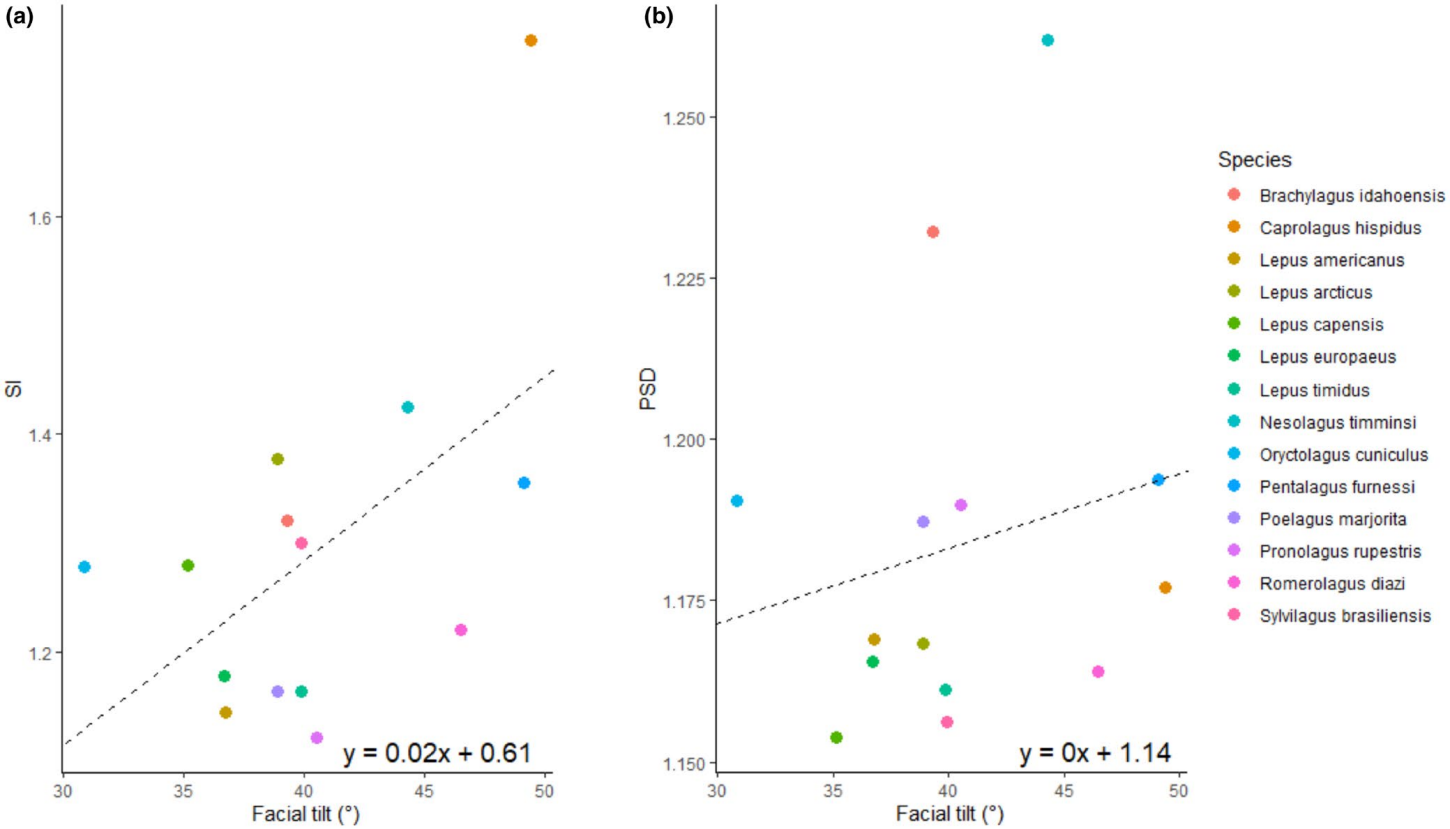
Plot showing the relationship between the complexity metrics and facial tilt. (a) SI and facial tilt. (b) PSD and facial tilt.

To compare complexity with facial tilt angle, we conducted a PGLS analysis where complexity scores were coded as the response variables and modelled as a function of the predictor variable facial tilt (Figure 3). Where SI is the response variable, the PGLS shows a significant relationship (SE = 0.0065, *t* = 3.2306, *p* = 0.0072; Figure 3a). In contrast, where PSD is the response variable, the PGLS shows no significant relationship (SE = 0.0012, *t* = 0.3222, *p* = 0.7528; Figure 3b).

### 3.4 Shape

**FIGURE 4:**
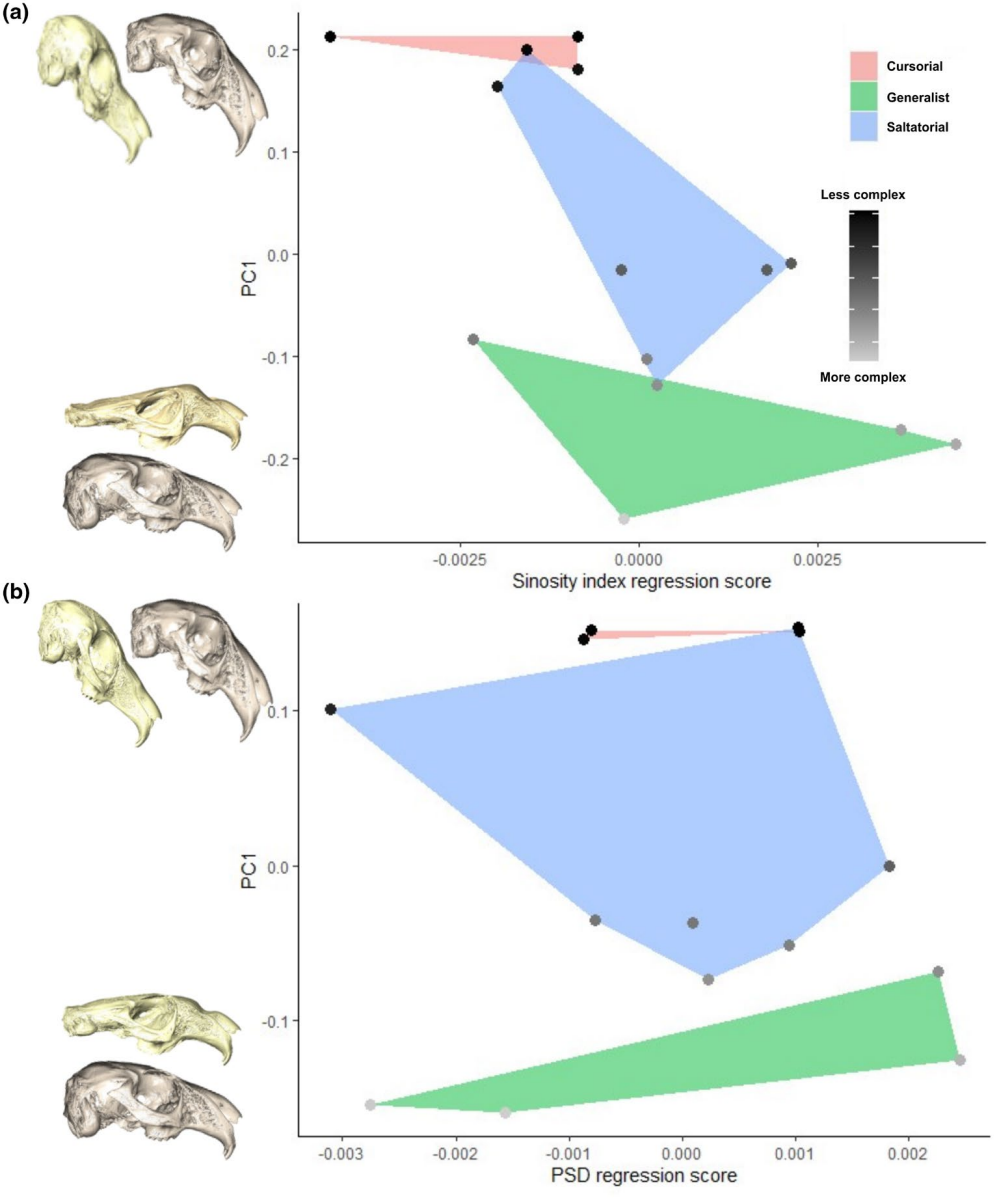
Plot showing the relationship between shape and complexity. Points are coloured on a gradient of most complex to least complex. Hulls represent locomotor modes. (a) SI and shape. (b) PSD and shape. The accompanying warped skulls are skulls warped from the PC1 of the shape–complexity PGLS (first) and PC1 of a shape only PCA (second). PGLS skulls appear extensively warped due to the regression of the data points. However, they show the general trend: Species with more complex intracranial joints tend to have doming of the skull and more acute facial tilting.

Procrustes superimposed coordinates were included in a PGLS to compare complexity with overall skull shape. We conducted separate phylogenetic generalised least square analyses where the two‐complexity metrics were the predictor variables and shape was the response. Where SI is the predictor variable, the PGLS shows a significant relationship (*r* ^2^ = 0.4790, *p* = 0.0053; Figure 4a). However, where PSD is the predictor variable, the PGLS result is not significant (*r* ^*2*^ = 0.1843, *p* = 0.0781; Figure 4b).

In the PGLS that includes SI values, principal component 1 for fitted values accounts for 80.76% of variance and principal component 2 for fitted values accounts for 8%. PC1 shape change is largely associated with expansion and reduction of the brain case, with significant doming of the skull at the positive extreme of PC1. When the SI regression score is plotted against PC1, the cursors occupy a region of morphospace that is characterised by positive PC1 and negative SI regression score. Species occupying the positive extreme PC1, with doming of the skull, tend to have lower SI scores and thus are deemed less complex.

In the PGLS that includes PSD values, principal component 1 for fitted values accounts for 68.45% of variance, and principal component 2 for fitted values accounts for 15.47%. The relationship between shape and PSD is not significant. This may be because a significant portion of shape difference appears to relate to changes associated with facial tilting. Since variation in sinuosity of the joint is also significantly related to facial tilt, but PSD is not, it is likely that the pattern seen in the shape and PGLS regression is largely driven by facial tilt angle.

### 3.5 Body mass and geometric size

To compare complexity with body size, we ran a PGLS model where complexity scores were coded as the response variables and modelled as a function of body mass, the predictor variable (see Figure S2A,B). For both SI and PSD, the PGLS indicates no significant relationship (SI: SE <0.0001 *t* = 1.8137, *p* = 0.0948, PSD: SE < 0.0001, *t* = −1.2884, *p* = 0.2219).

We also tested the relationship between complexity and cranial centroid size (see Figure S2C,D). For both SI and PSD, PGLS also indicates no significant relationship (SI: SE <0.0001 *t* = −1.4870, *p* = 0.1628, PSD: SE = 0.0019, *t* = 1.5281, *p* = 0.1524).

### 3.6 Burrowing

For each complexity metric, a one‐way ANOVA was performed to compare the effect of burrowing on complexity (see Figure S2E,F). For both SI and PSD, there was no statistically significant difference in scores between burrowing habit categories (*F* = 0.0320, *p* = 0.8620 and *F* = 0.6860, *p* = 0.4240 respectively).

## 4 DISCUSSION

The morphology, or complexity, of the ICJ in leporid lagomorphs has never been quantified or statistically correlated with functional traits, limiting our understanding of its evolution and function. Here, we use two‐complexity metrics to quantify the lambdoidal suture, which forms the dorsal portion of the ICJ and is hypothesised to act as a hinge for joint movement. The relationship between the SI complexity metric and locomotor mode was significant, but the relationship between the PSD complexity metric and locomotor mode was not. We found lower SI complexity in cursorial species, partially confirming our first hypothesis that complexity varies between locomotor modes. Namely, the aspects of complexity that the SI metric is sensitive to (particularly rate of interdigitation) vary between locomotor modes. Having a complex interdigitated ICJ may hinder the mobility of the suture, whereas a simpler, defined hinge may allow for greater motion between the two portions of the cranium. Increased definition of an intracranial hinge, associated with greater motion at that joint, is seen in psittacine birds (parrots), which have markedly more defined cranio‐facial hinges than other prokinetic birds (Tokita, 2003). Our findings provide more evidence for the long‐standing hypothesis that the ICJ is facilitating kinesis (Bramble, 1989). Furthermore, the rate of fusion in the bones surrounding the lambdoidal suture also results in a more defined, straight‐line hinge in the hares. In hares, the interparietal suture is obliterated (Bramble, 1989), which, combined with the lack of interdigitation in the ICJ, allows for a well‐defined patent hinge between the parietals and occipital.

We also found a significant relationship between the SI complexity metric and facial tilt angle, but not the PSD metric. This partially supports our second hypothesis that complexity covaries with facial tilt angle. SI is sensitive to interdigitation; essentially, we see less interdigitation in the joints of species with greater facial tilting (i.e., a more acute angle between the upper diastema and occipital plane). A greater facial tilt has been correlated with faster locomotion (Kraatz et al., 2015), which links our first and second hypotheses. Specifically, fast‐running cursorial species have greater facial tilt and a less complex ICJ. Both traits may be independently linked to locomotion, where ventral facial tilting allows greater frontation of the orbits, whilst the ICJ allows dissipation of kinetic energy. Alternatively, the ICJ and facial tilting may be linked into a functional unit, where the ICJ suture is left patent into adulthood to allow further advancement or ventral flexion of the anterior cranial unit.

This relationship between ICJ complexity (SI), facial tilt, and locomotor mode may therefore be tied to craniofacial growth during ontogeny. The most cursorial species of leporid, *Lepus*, are born precocial, and it has been demonstrated that hares possess decreasing locomotor mechanical advantage with increasing size and age (i.e., infant and juvenile hares have up to two times greater acceleration than adults at much lower body weight, contrary to the patterns found in other mammals (Carrier, 1995)). Because juvenile hares exhibit adult locomotor patterns, it may be functionally important for the lambdoidal suture, which makes up the dorsal part of the ICJ, to stay open into adulthood. This could help dissipate mechanical forces and reduce developmental stress on the brain and sensory systems (Bramble, 1989). However, aside from the European rabbit, there is a lack of literature on the ontogenetic growth of the craniofacial skeleton in leporids, especially precocial species such as those in the genus *Lepus*. In addition, the lambdoidal suture is of an unknown developmental origin (in a rodent model) and therefore data on the very early stages of development of the ICJ are missing (Lenton et al., 2005). More data on morphological changes during development and ontogeny in precocial species from the genus *Lepus* may provide more insight into the relationship between facial tilting and ICJ complexity at the lambdoidal suture.

Furthermore, the ICJ sits at the anatomical and developmental boundary between bones of different embryological origins. The anterior skull bones (such as the parietals) primarily undergo intramembranous ossification, and the posterior bones (such as the occipital) primarily develop through endochondral ossification (Kyomen et al., 2023). The placement of the ICJ at this boundary may contribute to its distinct mechanical and functional properties. Further work exploring the ontogeny of the joint, especially in precocial leporid species, could help clarify how these distinct ossification processes contribute to ICJ morphology, mechanical function, and variation across locomotor and ecological niches.

In addition, it is difficult to make inferences about locomotor performance and the effect on morphology across leporids because appropriate locomotor performance data is lacking at present. Previous studies in lagomorphs (Martin et al., 2022) and mammals more generally (Christiansen, 2002) have shown that certain anatomical measurements and ratios (e.g., metatarsal‐femoral ratio) may be moderately correlated with maximal running speeds. Though limb anatomy is shaped by multiple functional and phylogenetic processes, and is unlikely to tightly correlate with any single performance metric (Christiansen, 2002; Martin et al., 2022), the creation of performance proxies based upon these principles may be an improvement upon the discrete locomotor categorisation used here, whilst avoiding the need to acquire field‐based speed estimates which are challenging to attain reliably.

We found no relationship between the complexity of the ICJ and burrowing behaviour in leporids, as predicted by our third hypothesis. Whilst burrowing is documented to be related to cranial transformations in both rodents (Gomes Rodrigues et al., 2016) and lagomorphs (Todorov et al., 2022; Wake et al., 1993), leporid burrowing is limb‐dominated, as opposed to the chisel‐tooth digging behaviour seen in some rodents, which is associated with more extreme changes to skull morphology (Gomes Rodrigues et al., 2016; McIntosh & Cox, 2016a, 2016b). However, more robust data on the nature of burrowing is needed for leporids; there is often little distinction between those that actively burrow and those that utilise the burrows of other animals (Kraatz et al., 2015). Size (both body mass and centroid size for the skull) was also non‐significant in relation to complexity in the respective PGLS performed for these two variables. This result could be related to the extrinsically constrained size of leporids in general, thought to be associated with the close niche overlap with small ungulates (Tomiya & Miller, 2021).

## 5 CONCLUSIONS AND FUTURE WORK

In this study, we explored the complexity of the ICJ in leporid lagomorphs and its potential relationship to locomotor mode, cranial shape, facial tilt, body mass, cranial size, and burrowing habit. Our analyses revealed a significant relationship between ICJ complexity (SI), facial tilt, cranial shape, and locomotor mode. Specifically, cursorial species have greater facial tilt and less complex ICJs compared with those with scrambling or hopping‐based locomotion. A simple joint could facilitate more hinge‐like mobility, supporting the hypothesis that the ICJ could reduce the impact from high‐speed running on the cranium by providing intracranial movement (Bramble, 1989). However, further research is needed to better understand the mechanical properties of the joint and the forces experienced by the cranium during locomotion.

Testing this in live animals may not be feasible or ethical due to the limitations of measuring ICJ movement during treadmill running, specifically the difficulty of achieving truly saltatory running with strain gauges and electromyography for muscle activation. However, assessing the movement of the joint in cadaveric material may be possible using X‐ray reconstruction of moving morphology or digital volume correlation. In silico functional testing with finite element modelling could elucidate function in different parts of the cranium when simulating feeding and locomotion (Watson et al., 2021; Wood‐Bailey & Sharp, 2025). In addition, where current complexity analysis techniques are limited in their application due to the reduction of data to two dimensions, finite element modelling would enable the user to test the functional implications of variation in suture morphology in 3D. Histological investigation could be useful to develop a clearer picture of function in the joint by revealing tissue types present within the joint, such as elastin, fibrin, and collagen (Herring & Teng, 2000), and provide information on the proportions of fibre types present. Furthermore, a thorough understanding of the development of facial tilting and the ICJ complex is needed to determine their relationship to one another. Finally, any future work on the topic should expand the sample size to encompass a broader range of species (where possible) and it is recommended that a thorough examination of the rate of intraspecific variation in leporid skulls is undertaken. Nevertheless, our work, which quantifies the complexity of the leporid ICJ for the first time, identifies a relationship with locomotor mode and highlights the need for further work on the trait.

## Supporting information


Data S1.

Table S1.

Figure S1.

## Data Availability

Data openly available in a public repository that does not issue DOIs. The data that support the findings of this study are openly available in [Morphosource] at [https://www.morphosource.org/projects/000581975?locale=en].
