# Supplementary material for: Factors impacting the complexity of the leporid intracranial joint
Source: J Anat. 2025 Aug 7;248(1):28–39. doi: 10.1111/joa.70031 (PMC12682590; doi:10.1111/joa.70031)
Supplement: Supplementary file 2 — Table S1. [file JOA-248-28-s002.docx]

**Table 1. Genera and species included in the present study.**

| Species | Source | Accession number | Repository | Scan resolution |
| --- | --- | --- | --- | --- |
| *Lepus americanus* | AMNH | amnh: mammals:97648 | [Morphosource](https://www.morphosource.org/concern/media/000055349?locale=en) | 0.04x0.04x0.04 |
| *Brachylagus idahoensis* | AMNH | amnh: mammals:92869 | [Morphosource](https://www.morphosource.org/concern/media/000054441?locale=en) | 0.03x0.03x0.03 |
| *Lepus arcticus* | AMNH | amnh: mammals:42139 | [Morphosource](https://www.morphosource.org/concern/media/000055618?locale=en) | 0.05x0.05x0.05 |
| *Oryctolagus cuniculus* | AMNH | amnh: mammals:34816 | [Morphosource](https://www.morphosource.org/concern/biological_specimens/000S14432?locale=en) | 0.04x0.04x0.04 |
| *Poelagus marjorita* | AMNH | amnh: mammals:51052 | [Morphosource](https://www.morphosource.org/concern/media/000055707?locale=en) | 0.04x0.04x0.04 |
| *Caprolagus hispidus* | SMNH | A585848 | - | 0.12x0.12x0.12 |
| *Lepus capensis* | NML | NML-VZ 19.8.1875.29 | [Morphosource](https://www.morphosource.org/concern/media/000590591?locale=en) | 0.12x0.12x0.12 |
| *Lepus europaeus* | University of Liverpool | UoL: 2021.161140 | [Morphosource](https://www.morphosource.org/concern/media/000590600?locale=en) | 0.12x0.12x0.12 |
| *Lepus timidus* | University of Liverpool | UoL: 2021.1630203 | [Morphosource](https://www.morphosource.org/concern/media/000590608?locale=en) | 0.12x0.12x0.12 |
| *Pronolagus rupestris* | NML | NML-VZ A20.11.1908.3 | [Morphosource](https://www.morphosource.org/concern/media/000590618?locale=en) | 0.12x0.12x0.12 |
| *Sylvilagus brasiliensis* | NML | NML-VZ 1980.354 | [Morphosource](https://www.morphosource.org/concern/media/000590624?locale=en) | 0.12x0.12x0.12 |

**Table 2. 3D landmarks used to quantify shape variation in the leporid skull.** Adapted from Kraatz et al., (2016).

| 1 | Opisthion - Midline point on the dorsal margin of the foramen magnum |
| --- | --- |
| 2 | Basion - Midline point on the ventral margin of the foramen magnum |
| 3 | Anteriormost point of basioccipital along midsagittal line |
| 4 | Anteriormost point of the basisphenoid along midsagittal line |
| 5 | Staphilion - Midline point on the anterior choanal margin on the hard palate. In the leporid condition, this condition changes from two parasagittal concavities (opening caudally), to a singular concavity with age. In the presence of two concavities, the landmark is marked at the point that intersects a line drawn laterally between the anterior most point of both concavities, and the midsagittal line. |
| 6 | Midline posterior margin of incisive foramina; measured similarly to landmark 5. |
| 7 | Anteriormost point of incisive foramen in midsagittal line |
| 8 & 30 | Posteriormost point of alveoli of I3 |
| 9 & 32 | Posterior extreme of alveolar margin of cheek tooth row |
| 10 & 31 | Anterior extreme of alveolar margin of cheek tooth row |
| 11 | Rhinion – Anterior-most point on nasal along midsagittal line |
| 12 | Nasospinale - Inferiormost point of nasal opening (premaxilla) |
| 13 & 22 | Posteriormost point of nasal on skull roof |
| 14 & 23 | Posterior extent of the premaxilla on skull roof |
| 15 & 26 | Anteriormost point of the posterior side of supraorbital process |
| 16 & 27 | Postero-lateral extent of the supraorbital process |
| 17 & 25 | Anteriormost point of posterior margin of the maxillary root of the zygomatic arch |
| 18 & 28 | Posteriormost point of the posterior margin of the squamosal root of the zygomatic arch |
| 19 & 29 | Posterior extent of the posterior projecting jugal process of the zygomatic arch |
| 20 & 24 | Anteroventral-most point of masseteric spine |
| 21 | Chiasmatic sulcus - Point of contact between anterodorsal optic canals and presphenoid along midsagittal line as optic nerve emerges from braincase |
| 33 & 34 | Lateralmost point of the magnum foramen |
| 35 & 36 | Dorsalmost point of the articular surface of the occipital condyle |
| 37 & 38 | Medialmost margin of hypoglossal foramen |
| 39 | Posteriormost point of the external occipital protuberance in the sagittal plane |
| 40 | Bregma - Posteriormost point of inter-nasal suture |
| 41 | Midsagittal point on parietal-frontal suture |
| 42 & 43 | Ventralmost point of the petrosal bar of the squamosal |
