# Supplementary material for: Factors impacting the complexity of the leporid intracranial joint
Source: J Anat. 2025 Aug 7;248(1):28–39. doi: 10.1111/joa.70031 (PMC12682590; doi:10.1111/joa.70031)

**Supplementary figure 1. Phylogenetic tree of extinct and extant lagomorphs and close relatives.** Tupaia are used as the out-group. This relaxed-clock phylogenetic reconstruction was derived from morphological data (Wood-Bailey et al., 2022). The model was supplemented with divergence information derived from previously published molecular phylogenies (Ge et al., 2013).


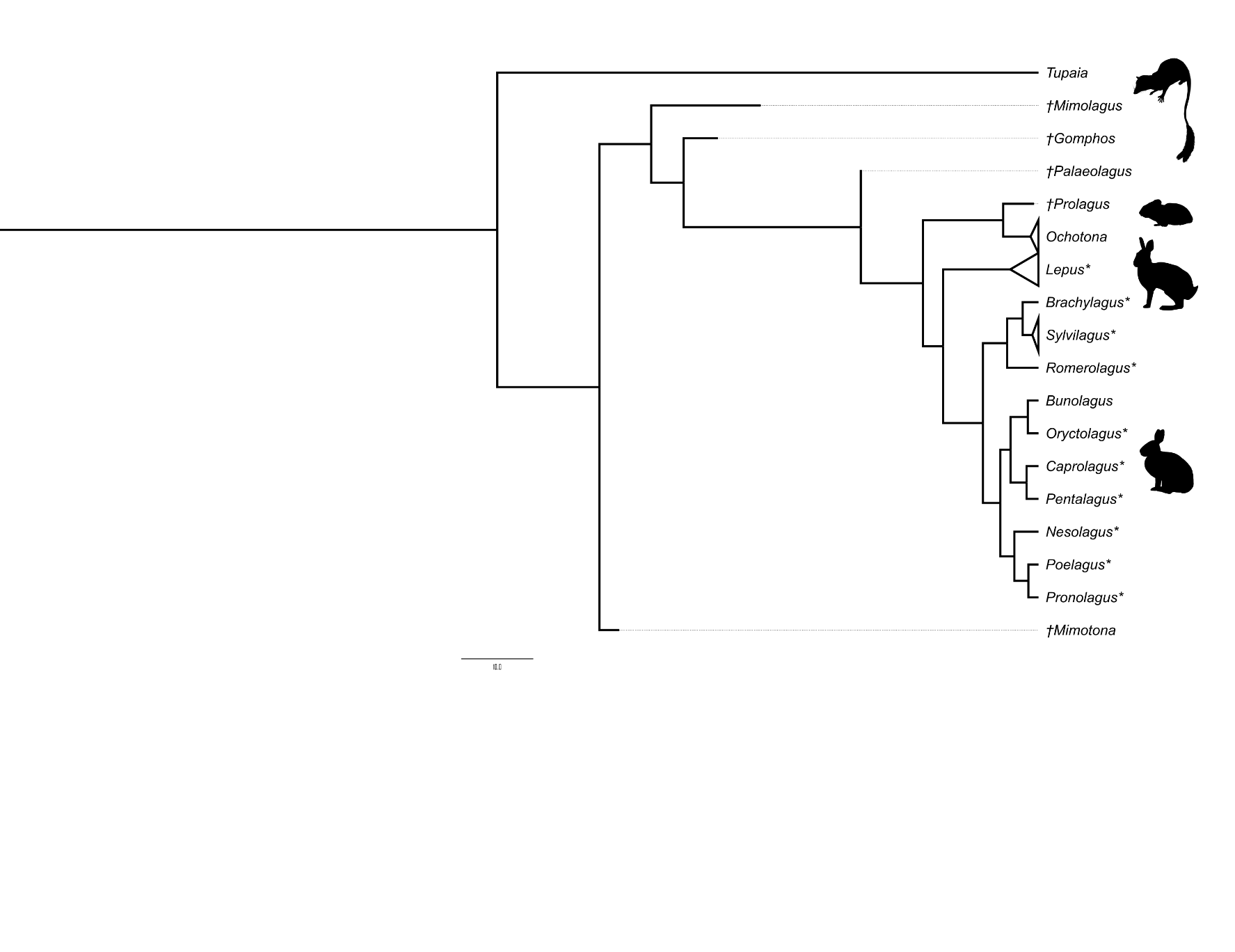


**Supplementary figure 2. Plots for non-significant tests: complexity and mass, centroid size and burrowing.** PGLS: A. Body mass and SI, B. Body mass and PSD, C. Centroid size and SI, D. Centroid size and PSD, ANOVA: E. Burrowing and SI, F. Burrowing and PSD.


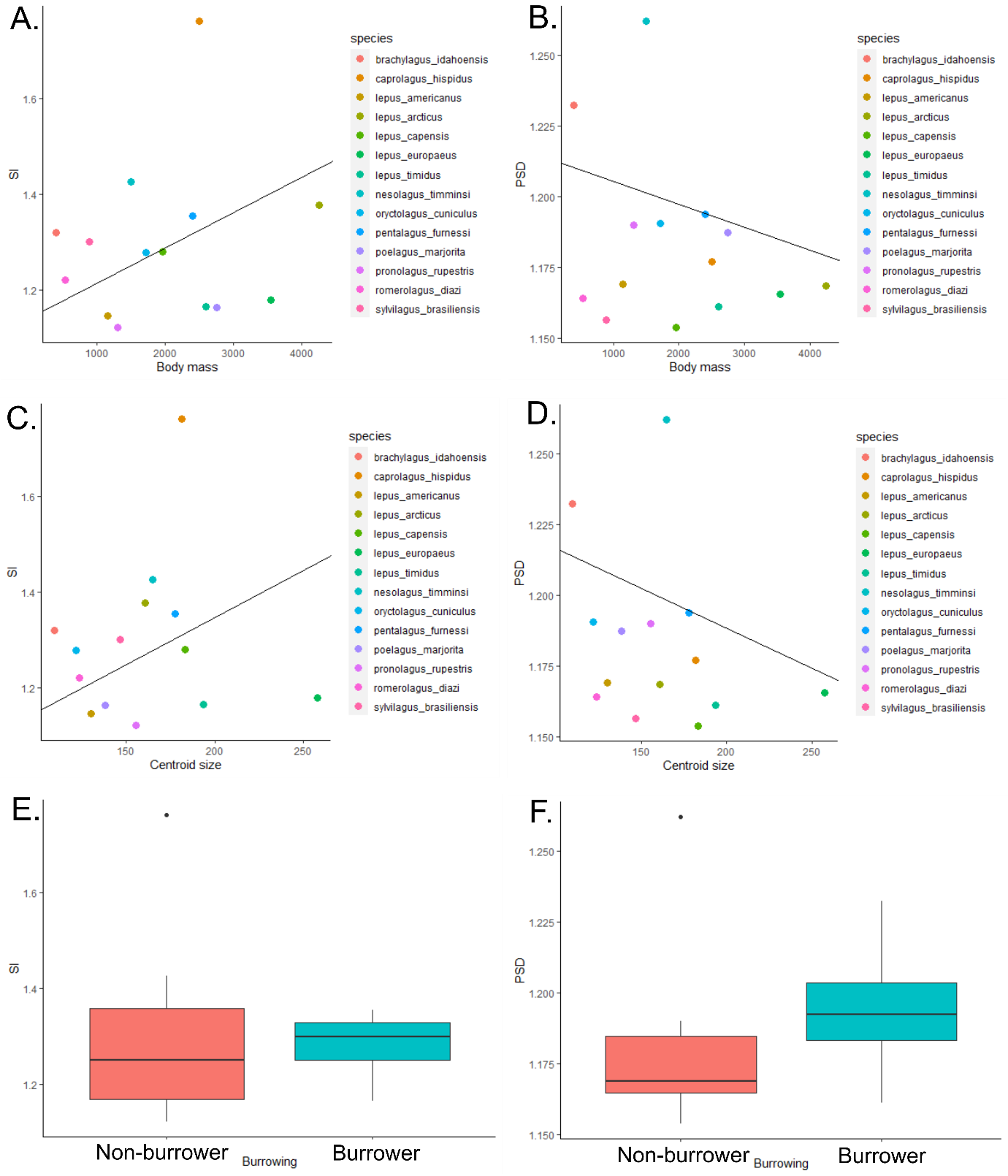

Supplement: Supplementary file 3 — Figure S1. [file JOA-248-28-s001.docx]
